# [Corrigendum] Temozolomide abrogates the aggressiveness of urothelial carcinoma cells by enhancing senescence and depleting the side population

**DOI:** 10.3892/ol.2026.15710

**Published:** 2026-06-17

**Authors:** Na-Yon Kim, Sung-Hyun Hwang, Yeseul Yang, Yongbaek Kim

Oncol Lett 22: 845, 2021; DOI: 10.3892/ol.2021.13106

Following the publication of the above paper, it was drawn to the authors’ attention by an interested reader that, regarding the flow cytometric experiments in Fig. 1C on p. 3, the ‘T24/CML’ and ‘J82/TMZ’ plots were apparently matching, suggesting that this figure had been assembled incorrectly. In addition, concerning the colony formation assay data in Fig. 1B, the ‘T24/0 μM’ and ‘J82/20 μM’, and ‘J82/0 μM’ and T24/20 μM’ pairings of images were apparently the same. Furthermore, upon performing an independent analysis of the data in this paper in the Editorial Office, it came to light that, regarding the scratch-wound assay images shown in Fig. 1D, the J82/0 h/T24 panel contained a small overlapping section with the T24/0 h/TMZ panel, such that these data panels were derived from the same original source, where different experimental conditions were described. In addition, it also came to light that the data panel shown for the J82/CTL experiment in Fig. 3B was apparently matching with a data panel that was published in a paper in *Cancer Gene Therapy* in 2015 featuring the corresponding author (Yongbaek Kim) in common (entitled “Inhibition of hedgehog signaling reduces the side population in human malignant mesothelioma cell lines”).

Upon consulting their original data, the authors have realized that mistakes were made regarding the assembly of Figs. 1 and 3. The authors have repeated the colony formation assay experiments (Fig. 1B), the flow cytometric assay experiments (Fig. 1C) and the scratch-wound assay experiments (Fig. 1D), and provided the correct data panel for the J82/CTL experiment in Fig. 3B, and the revised versions of Figs. 1 and 3, featuring the revised data in Fig. 1B-D and in Fig. 3B, are shown on the next two pages. Although the results obtained in the repeated experiments are very similar to those of the original experiments, showing that the replacement of these data has not altered the overall conclusions reported in this study, the descriptions of the data in the Results for these figures should now be revised to the following (changes are highlighted in **bold**): For Fig. 1B and C: “Quantification of the cell fractions revealed apoptotic cells (Q2 + Q4) in **0.03±0.05%** of the control group and **0.02±0.03%** of the TMZ-treated T24 cells, and **0.05±0.05%** of the control group and 0.04±0.04% of the TMZ-treated J82 cells (Fig. 1C).” In addition, concerning the description of the results for Fig. 3B, the relevant text should now read as follows: “Similarly, TMZ treatment decreased the SP fraction of J82 cells from **0.8** to 0.3%.”

The authors are grateful to the Editor of *Oncology Letters* for allowing them the opportunity to publish this Corrigendum, and all the authors agree with its publication. They also thank the reader of the article for drawing these matters to their attention, and apologize to the readership for any inconvenience caused.

## Figures and Tables

**Figure 1.**
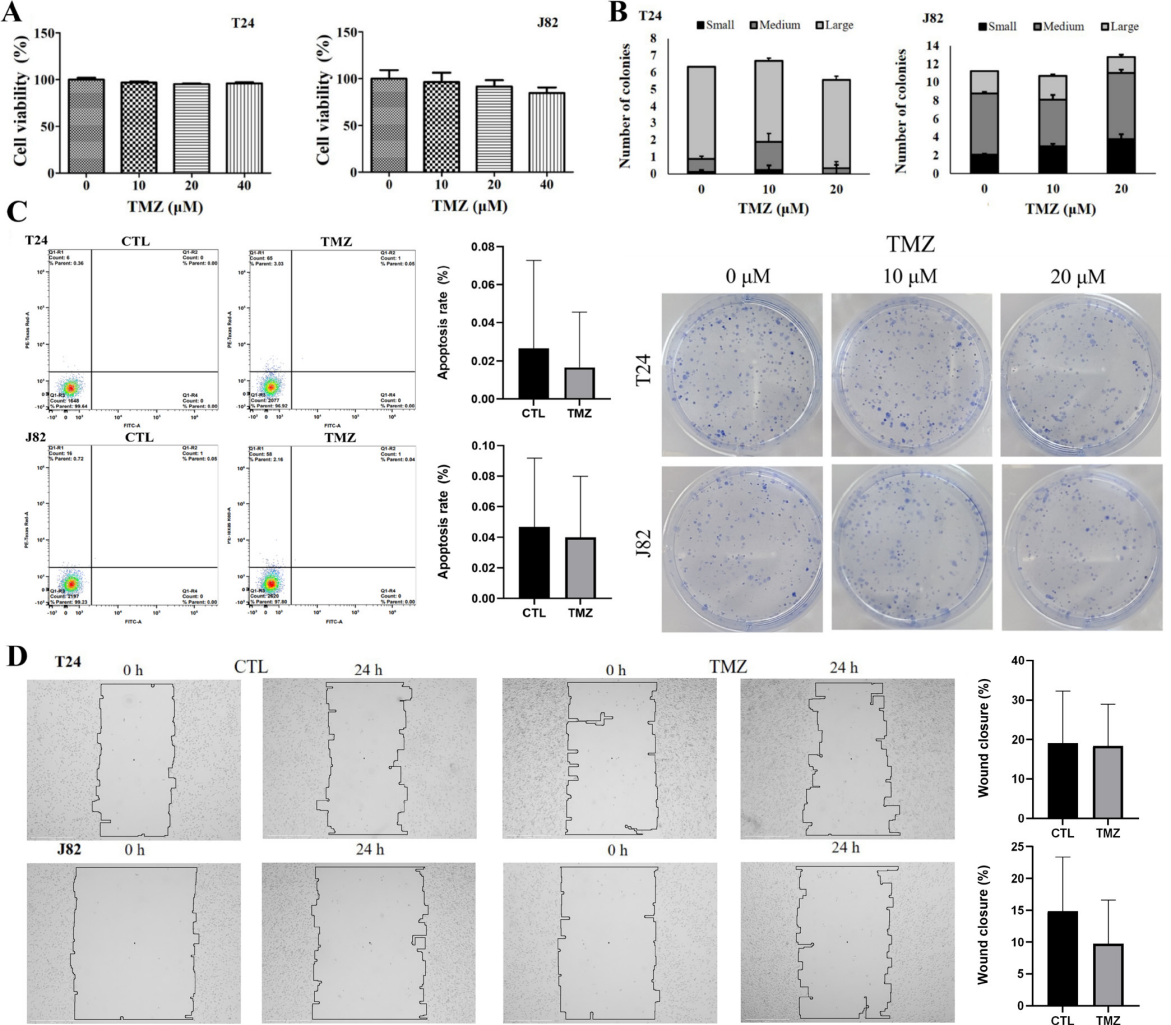
(A) Cell viability assay. TMZ at concentrations of 0, 10, 20 and 40 µM did not significantly alter the cell viability of human urothelial carcinoma cells. Data are presented as mean ± SD. (B) Colony-forming assay. TMZ did not significantly alter the colony-forming ability of human urothelial carcinoma cells. There were no significant changes in the total number or composition of the colonies. Colonies with 5–10, 11–50 and >50 cells were classified as small, medium and large, respectively. Each data point represents the mean ± SD. (C) Apoptosis assay. Results of fluorescence-activated cell sorting of urothelial carcinoma cells stained with Annexin V-FITC and propidium iodide are presented as a scatter diagram. (D) Scratch wound-healing assay. Relative cell migration showed that TMZ did not significantly affect the proliferation and migration capacity of human urothelial carcinoma cells. Black outlines indicate the initial wound area. Dotted lines indicate the edge of the wound after 24 h. CTL, control; TMZ, temozolomide; FITC, fluorescein isothiocyanate.

**Figure 3.**
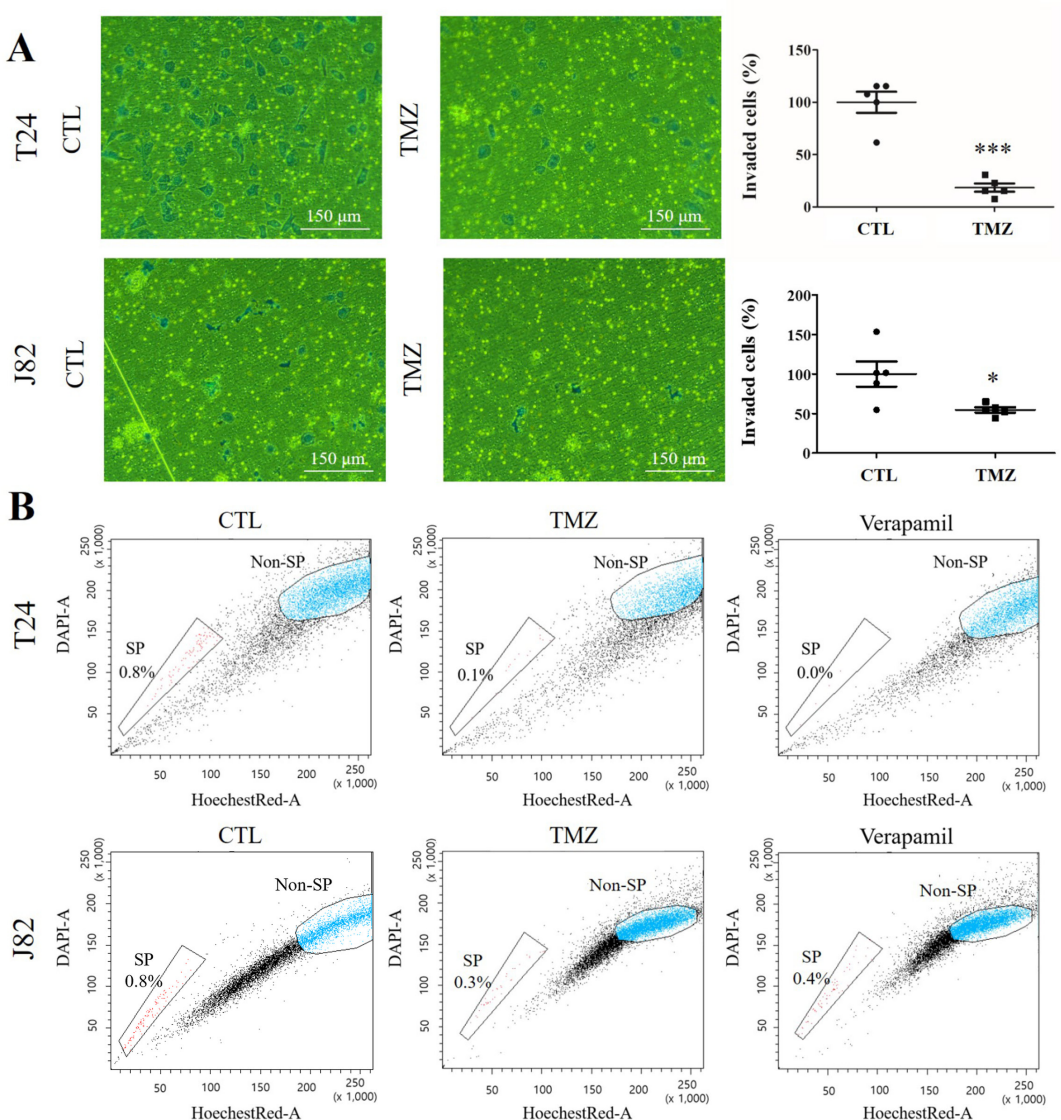
(A) Transwell migration assay. In the transwell migration assay, the invasiveness of TMZ-treated urothelial carcinoma cells was remarkably lower than that of untreated CTL cells. TMZ reduced the invasiveness of T24 cells to 18.46%±3.92% compared with the CTL group and that of J82 cells to 54.69%±3.40% compared with CTL group. *P<0.05; ***P<0.001. (B) SP assay. The SP fraction displayed a high dye efflux activity, which appeared as a clearly delineated tail in the lower left portion of the histogram. The SP was validated by verapamil treatment. Representative flow cytometry histograms are shown. TMZ treatment markedly reduced the side population fraction. SP, side population; CTL, control; TMZ, temozolomide.

